# Supplementary material for: Predicting future hospital antimicrobial resistance prevalence using machine learning
Source: Commun Med (Lond). 2024 Oct 10;4:197. doi: 10.1038/s43856-024-00606-8 (PMC11467333; doi:10.1038/s43856-024-00606-8)

Supplementary Methods

XGBoost parameters

Default XGBoost parameters as in main documentation
https://xgboost.readthedocs.io/en/stable/parameter.html:
eta=0.3, gamma=0, max_depth=6, min_child_weight=1, max_delta_step=0

Hyperparameter tuning grid search space:

```
  n_estimators = [10, 50, 100, 150, 200, 300, 400]
  max_depth = [1, 2, 3, 4, 5, 8, 12]
  min_child_weight = [3,5,7,10]

  param_grid = dict(max_depth=max_depth, n_estimators=n_estimators,
min_child_weight=min_child_weight)

  kfold = KFold(n_splits=3, shuffle=True, random_state=7)

  model = XGBRegressor(objective='reg:absoluteerror', seed=42)
  grid_search = GridSearchCV(model, param_grid, scoring="neg_mean_absolute_error",
n_jobs=-1, cv=kfold, verbose=1)
  grid_result = grid_search.fit(X_2021_np, y_2021_np)
  model_t =
XGBRegressor(**grid_search.best_params_,eval_metric=mean_absolute_error,seed=42)
  fit = model_t.fit(
    X_2021_np,
    y_2021_np,
verbose=True)
```

Tables

Supplementary Table 1 Number of Trust-pathogen-antibiotic-FYs that were excluded due to <=10 isolates tested by pathogen-antibiotic combination and the number of Trusts from which these FYs came

Pathogen-antibiotic combination	*Number of financial years excluded (N=5/6 FYs*119)*	*Number of trusts with excluded financial years (N=119)*
E.coli 3rd generation cephalosporins	18/714 (3%)	7
E. coli amoxicillin/clavulanic acid	27/714 (4%)	9
E. coli carbapenems	15/714 (2%)	6
E. coli ciprofloxacin	20/714 (3%)	7
E. coli gentamicin	14/714 (2%)	6
E. coli piperacillin/tazobactam	39/714 (5%)	13
Klebsiella sp. 3rd generation cephalosporins	22/595 (4%)	11
Klebsiella sp. amoxicillin/clavulanic acid	27/595 (5%)	11
Klebsiella sp. carbapenems	17/595 (3%)	9
Klebsiella sp. ciprofloxacin	22/595 (4%)	10
Klebsiella sp. gentamicin	14/595 (2%)	8
Klebsiella sp. piperacillin/tazobactam	37/595 (6%)	16
MSSA clarithromycin	437/714 (61%)	79
MSSA clindamycin	95/714 (13%)	30
MSSA erythromycin	120/714 (17%)	28
MSSA tetracycline	29/714 (4%)	15
MSSA vancomycin	144/714 (20%)	43
P. aeruginosa carbapenems	94/595 (16%)	40
P. aeruginosa ceftazidime	94/595 (16%)	39
P. aeruginosa ciprofloxacin	89/595 (15%)	39
P. aeruginosa gentamicin	123/595 (21%)	60
P. aeruginosa piperacillin/tazobactam	107/595 (18%)	44

Supplementary Table 2 Number of financial years that each Trust had >10 tested isolates, and hence were included in analyses, for each pathogen-antibiotic combination.

	Number of financial years contributing data						*Number of Trusts with any data*
Pathogen-antibiotic	**1**	**2**	**3**	**4**	**5**	**6**	
E. coli amoxicillin/clavulanic acid	1	1	3	0	3	110	118
E. coli carbapenems	1	1	1	0	3	113	119
E. coli 3rd generation cephalosporins	1	0	1	0	4	112	118
E. coli ciprofloxacin	1	0	2	0	3	112	118
E. coli gentamicin	1	0	2	0	3	113	119
E. coli piperacillin/tazobactam	1	0	5	2	3	106	117
Klebsiella sp. amoxicillin/clavulanic acid	2	0	2	5	108	/	117
Klebsiella sp. carbapenems	0	1	2	5	110	/	118
Klebsiella sp. 3rd generation cephalosporins	0	0	3	6	108	/	117
Klebsiella sp. ciprofloxacin	0	1	2	5	109	/	117
Klebsiella sp. gentamicin	0	0	2	5	111	/	118
Klebsiella sp. piperacillin/tazobactam	0	3	3	7	103	/	116
MSSA clarithromycin	3	5	2	2	2	40	54
MSSA clindamycin	2	4	3	4	10	89	112
MSSA erythromycin	0	3	4	0	6	91	104
MSSA tetracycline	1	0	4	2	8	104	119
MSSA vancomycin	3	4	9	4	12	76	108
P. aeruginosa carbapenems	5	7	5	18	79	/	114
P. aeruginosa ceftazidime	5	11	6	14	80	/	116
P. aeruginosa ciprofloxacin	3	12	5	16	80	/	116
P. aeruginosa gentamicin	4	9	17	26	59	/	115
P. aeruginosa piperacillin/tazobactam	3	14	6	16	75	/	114

Note: For *Klebsiella* sp. and *P. aeruginosa* a maximum of 5 FYs were available (indicated through a /).

Supplementary Table 3 Trusts with <100 isolates tested per financial year across all the pathogen-antibiotic combinations excluded from analyses

Trust	*Maximum number of tests per pathogen-antibiotic per financial year*	*Trust type*
MOORFIELDS EYE HOSPITAL NHS FOUNDATION TRUST	0	ACUTE - SPECIALIST
QUEEN VICTORIA HOSPITAL NHS FOUNDATION TRUST	0	ACUTE - SPECIALIST
AIREDALE NHS FOUNDATION TRUST	1	ACUTE - SMALL
NORTH TEES AND HARTLEPOOL NHS FOUNDATION TRUST	1	ACUTE - MEDIUM
THE ROYAL ORTHOPAEDIC HOSPITAL NHS FOUNDATION TRUST	3	ACUTE - SPECIALIST
ROYAL NATIONAL ORTHOPAEDIC HOSPITAL NHS TRUST	5	ACUTE - SPECIALIST
THE ROBERT JONES AND AGNES HUNT ORTHOPAEDIC HOSPITAL NHS FOUNDATION TRUST	7	ACUTE - SPECIALIST
LIVERPOOL HEART AND CHEST HOSPITAL NHS FOUNDATION TRUST	18	ACUTE - SPECIALIST
THE WALTON CENTRE NHS FOUNDATION TRUST	19	ACUTE - SPECIALIST
SHEFFIELD CHILDREN'S NHS FOUNDATION TRUST	21	ACUTE - SPECIALIST
ROYAL PAPWORTH HOSPITAL NHS FOUNDATION TRUST	22	ACUTE - SPECIALIST
LIVERPOOL WOMEN'S NHS FOUNDATION TRUST	23	ACUTE - SPECIALIST
ALDER HEY CHILDREN'S NHS FOUNDATION TRUST	24	ACUTE - SPECIALIST
THE CLATTERBRIDGE CANCER CENTRE NHS FOUNDATION TRUST	27	ACUTE - SPECIALIST
GREAT ORMOND STREET HOSPITAL FOR CHILDREN NHS FOUNDATION TRUST	32	ACUTE - SPECIALIST
BIRMINGHAM WOMEN'S AND CHILDREN'S NHS FOUNDATION TRUST	40	ACUTE - SPECIALIST
THE ROYAL MARSDEN NHS FOUNDATION TRUST	63	ACUTE - SPECIALIST
THE CHRISTIE NHS FOUNDATION TRUST	91	ACUTE - SPECIALIST
DORSET COUNTY HOSPITAL NHS FOUNDATION TRUST	97	ACUTE - SMALL

Supplementary Table 4 Number of Trusts with 0% resistance for all observed financial years for each specific pathogen-antibiotic combination. The pathogen-antibiotic combinations that are missing had 0 Trusts with 0% resistance throughout.

Pathogen-antibiotic	*Number of Trusts with resistance 0% throughout up to 2020-2021 (N=119)*	*Number of Trusts with resistance 0% throughout up to 2021-2022 (N=119)*
E. coli carbapenems	**69**	**62**
Klebsiella sp. carbapenems	**56**	**53**
Klebsiella sp. ciprofloxacin	1	0
Klebsiella sp. gentamicin	2	1
MSSA tetracycline	1	0
MSSA vancomycin	**93**	**91**
P. aeruginosa carbapenems	10	7
P. aeruginosa ceftazidime	12	9
P. aeruginosa ciprofloxacin	13	11
P. aeruginosa gentamicin	19	16
P. aeruginosa piperacillin/tazobactam	15	10

Figures

Supplementary Figure 1 Difference in resistance prevalence between current and previous year by number of tests per year for the specific Trust-pathogen-antibiotic combinations.

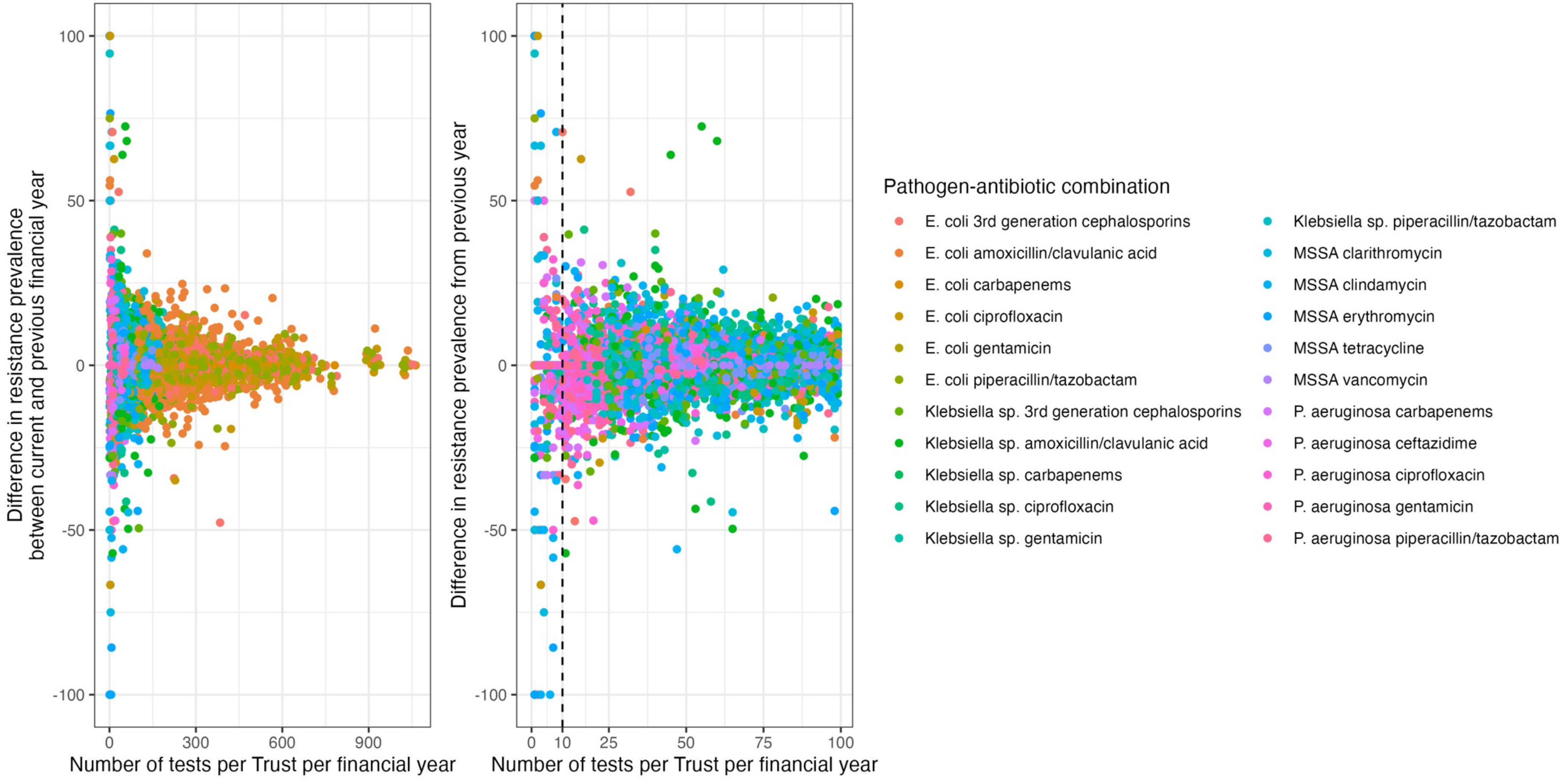

Note: one point per Trust-pathogen-antibiotic-financial year. Right-hand panel is a subset of the left-hand panel with the vertical black dashed line at the arbitrary threshold for inclusion (x=10).

Supplementary Figure 2 Distribution of resistance prevalence per pathogen-antibiotic across Trusts and financial years.

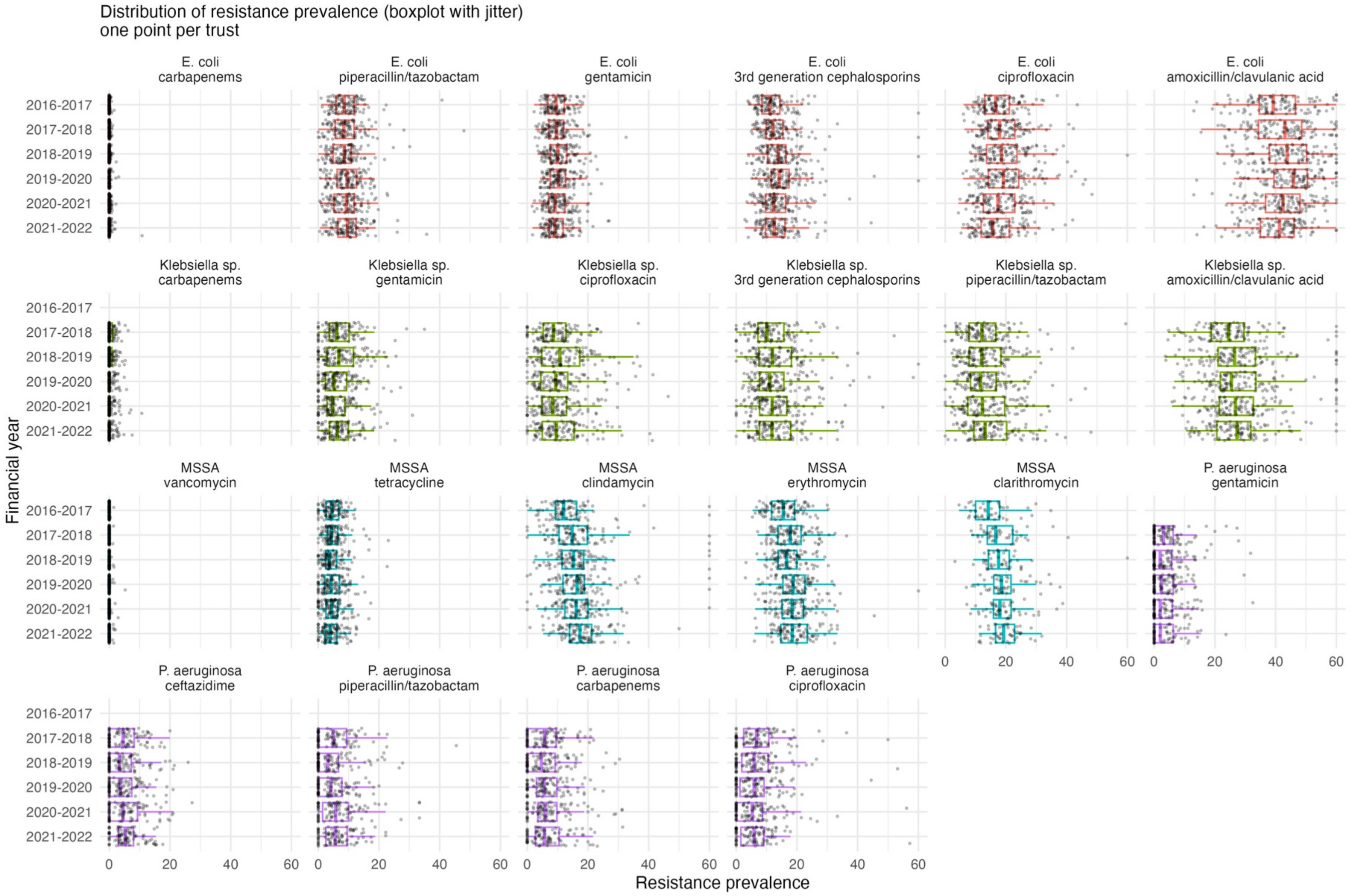

Note: one point per Trust. Outliers outside of x-axis scale (absolute value >10) were truncated. Center line, median; box limits, upper and lower quartiles; whiskers, 1.5x interquartile range.

Supplementary Figure 3 Distribution of difference between the current and previous financial year resistance prevalence, per pathogen-antibiotic across Trusts and financial years.

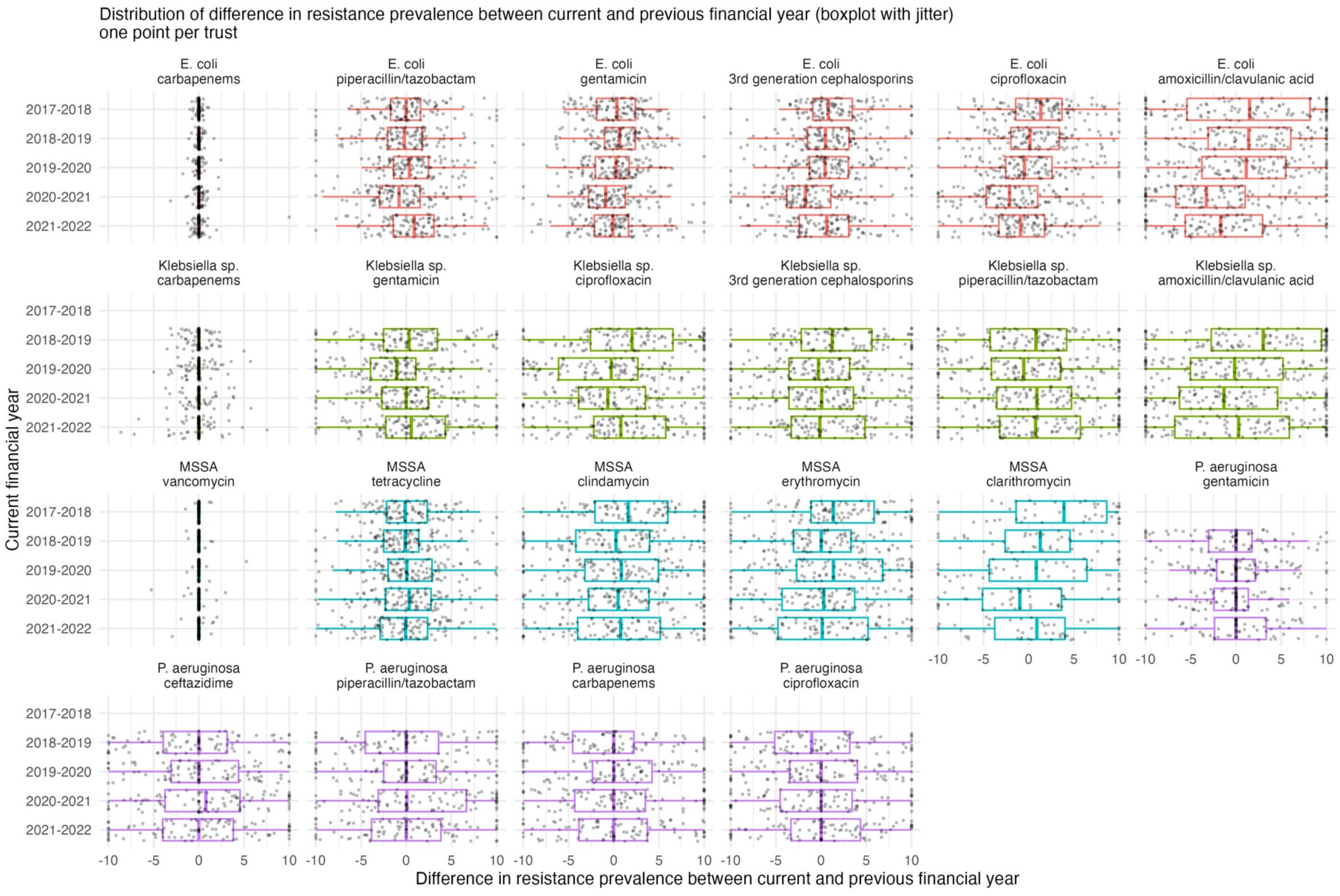

Note: one point per Trust. Outliers outside of x-axis scale (absolute value >10) were truncated. Center line, median; box limits, upper and lower quartiles; whiskers, 1.5x interquartile range.

Supplementary Figure 4 Percentage of Trusts with absolute difference between the current and previous financial year resistance prevalence <5%, <7.5%, and <10% per pathogen-antibiotic-FY.

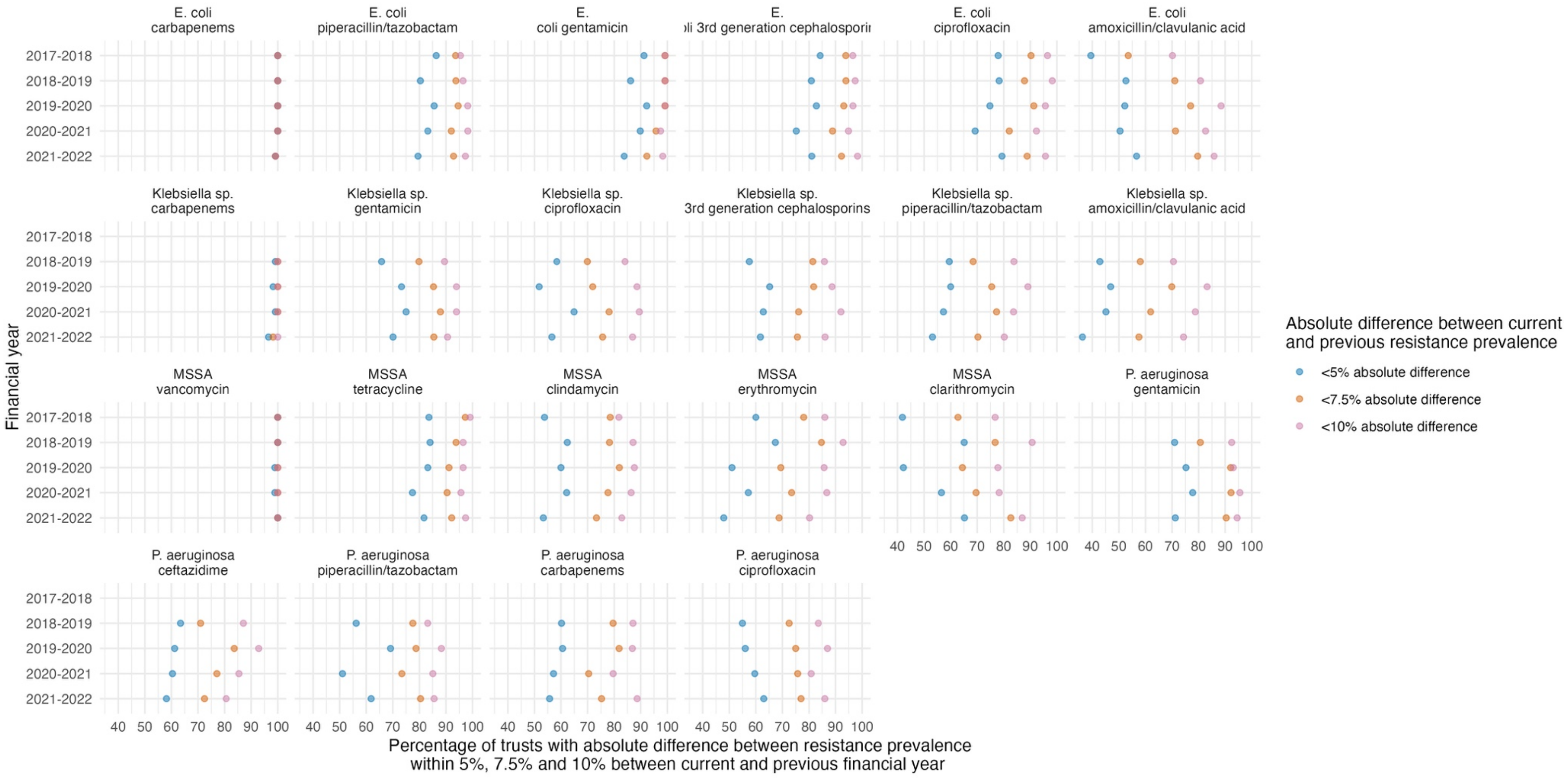

Supplementary Figure 5 Distribution of mean (A) and standard deviation (B) of antibiotic usage rate per antibiotic per Trust across available financial years.

(A)

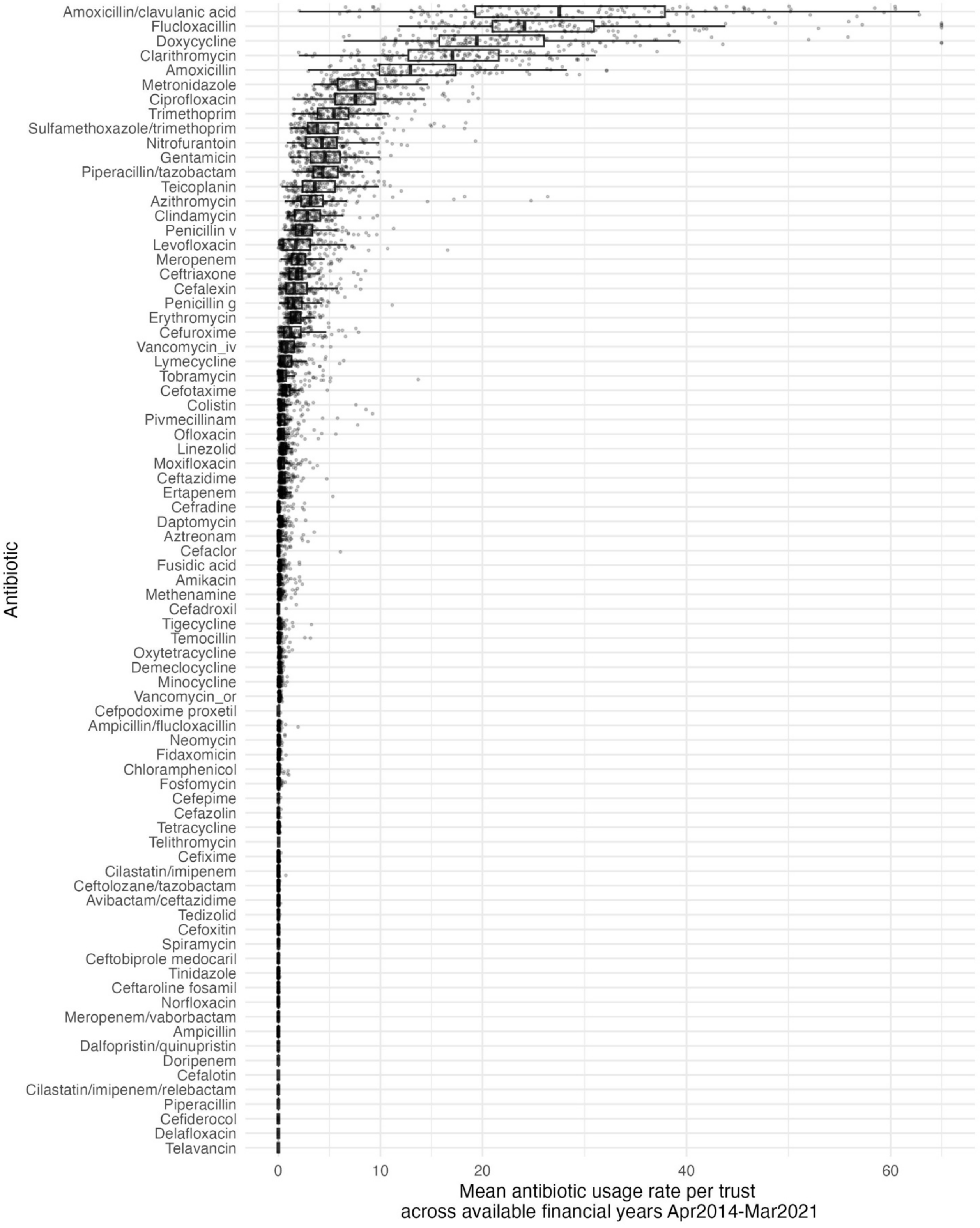

(B)

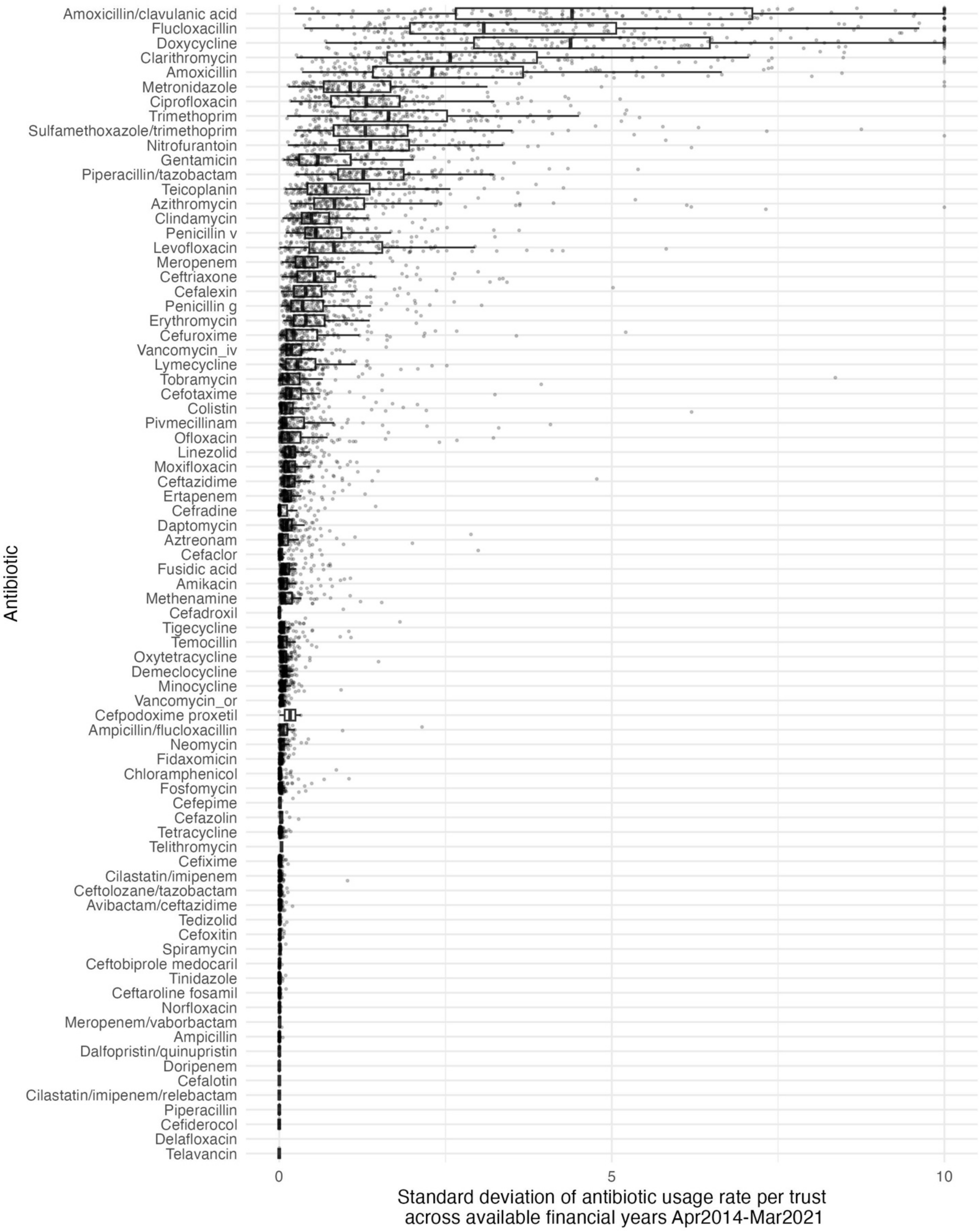

Note: Outliers outside of x-axis scale (absolute value >10) were truncated. Center line, median; box limits, upper and lower quartiles; whiskers, 1.5x interquartile range.

Supplementary Figure 6 Distribution of antibiotic usage per antibiotic across Trusts and financial years, for the top 24 most used antibiotics.

(A)

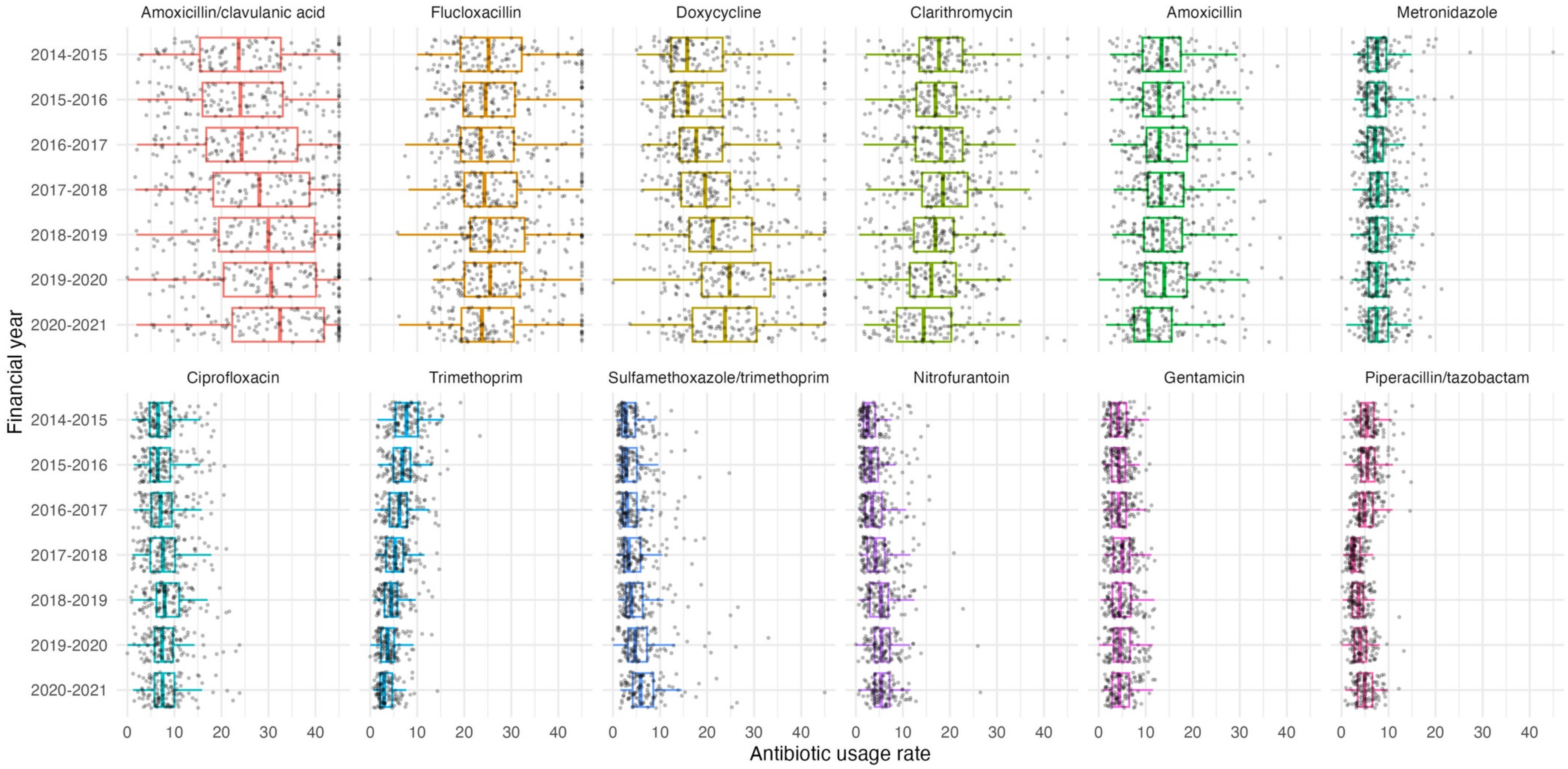

(B)

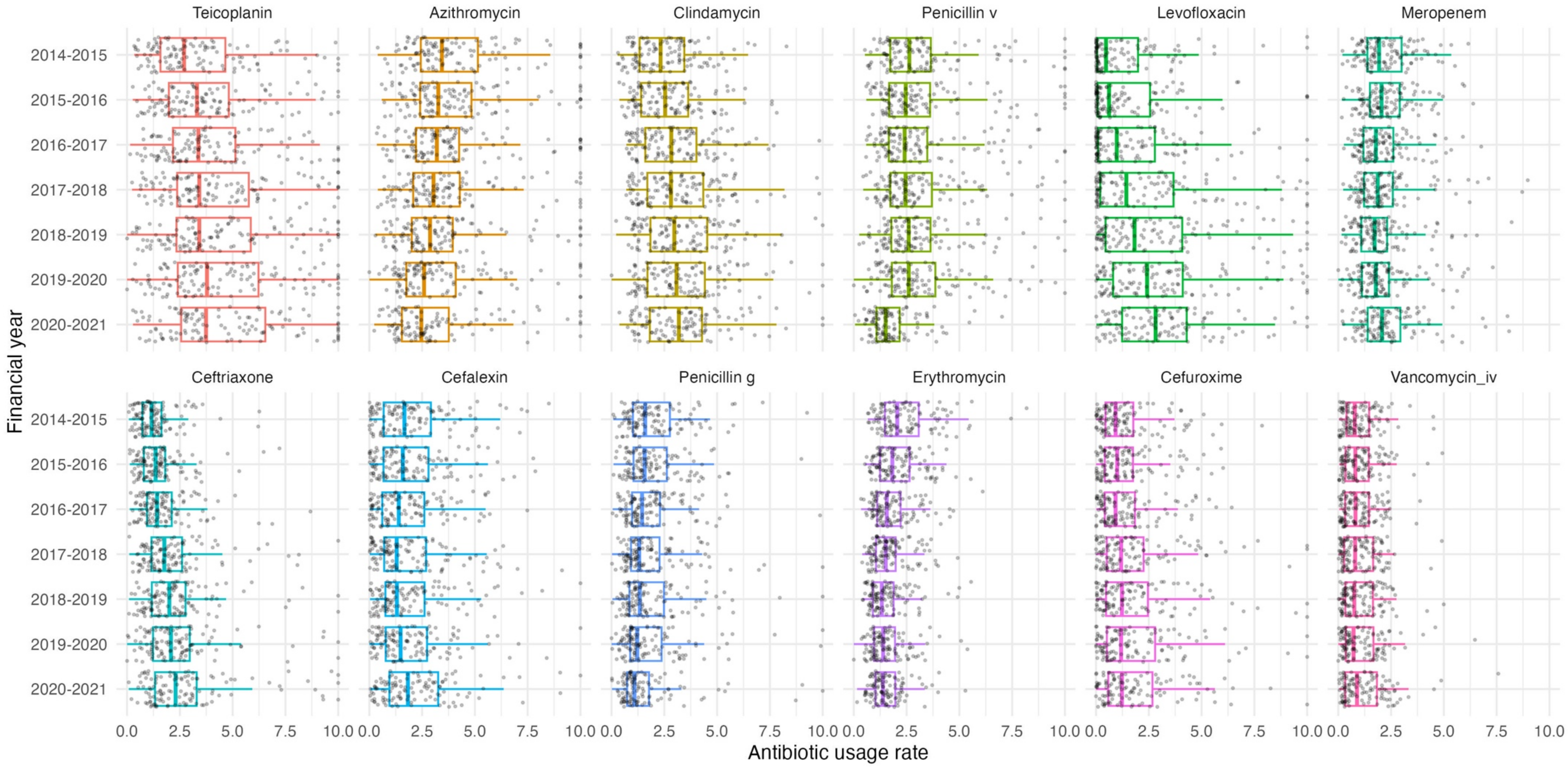

Note: Panel (A) and (B) have different scales on the y-axis. Outliers outside of x-axis scale (absolute value >10) were truncated. Center line, median; box limits, upper and lower quartiles; whiskers, 1.5x interquartile range.

Supplementary Figure 7 Distribution of difference between the current and previous financial year antibiotic usage rate per antibiotic across all Trust-FYs for the top 12 most used antibiotics.

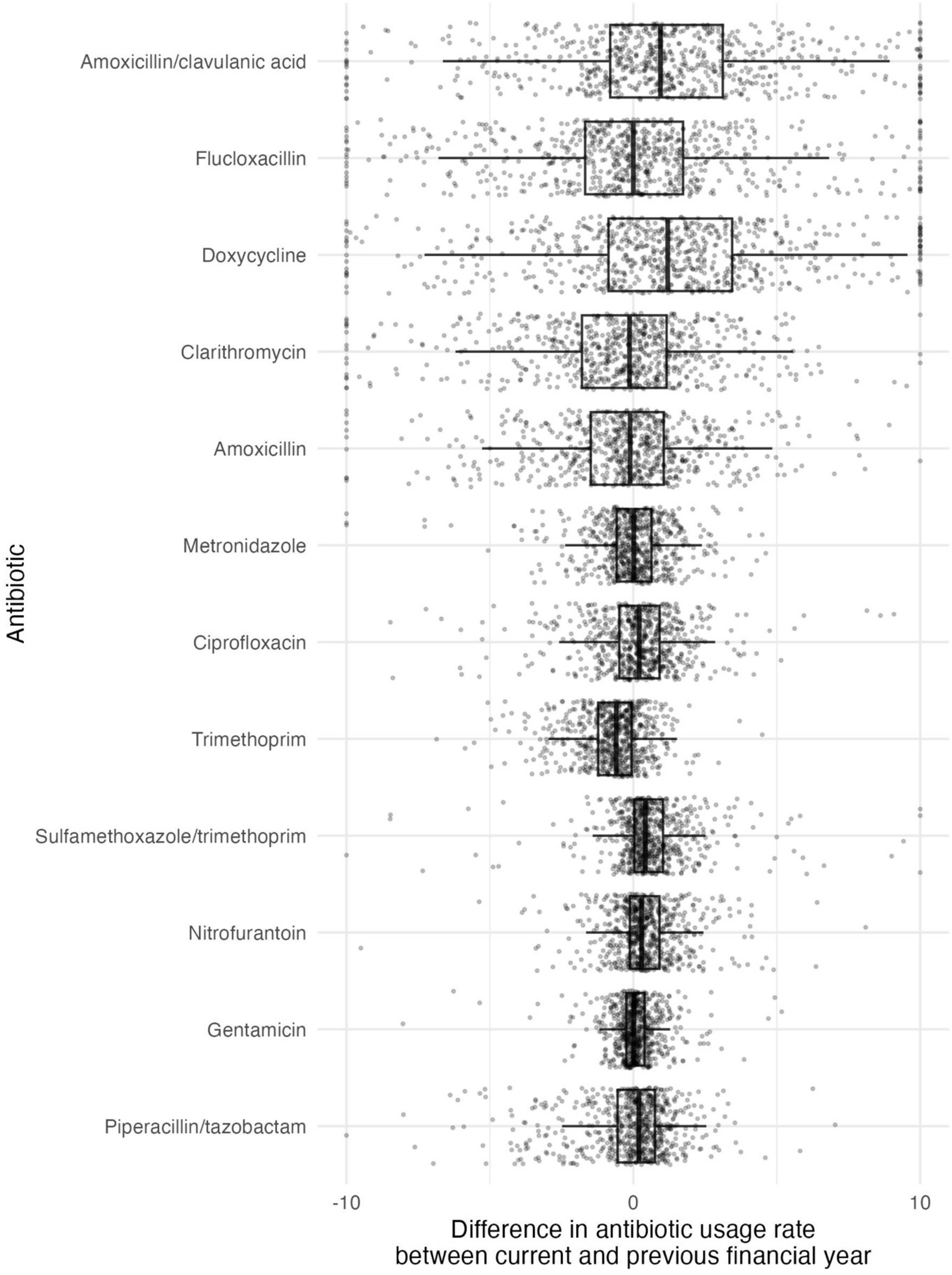

Note: one point per Trust-antibiotic-FY. Outliers outside of x-axis scale (absolute value >10) were truncated. Center line, median; box limits, upper and lower quartiles; whiskers, 1.5x interquartile range.

Supplementary Figure 8 Distribution of difference between the current and previous financial year antibiotic usage rate by financial years across all trusts for the top 12 most used antibiotics.

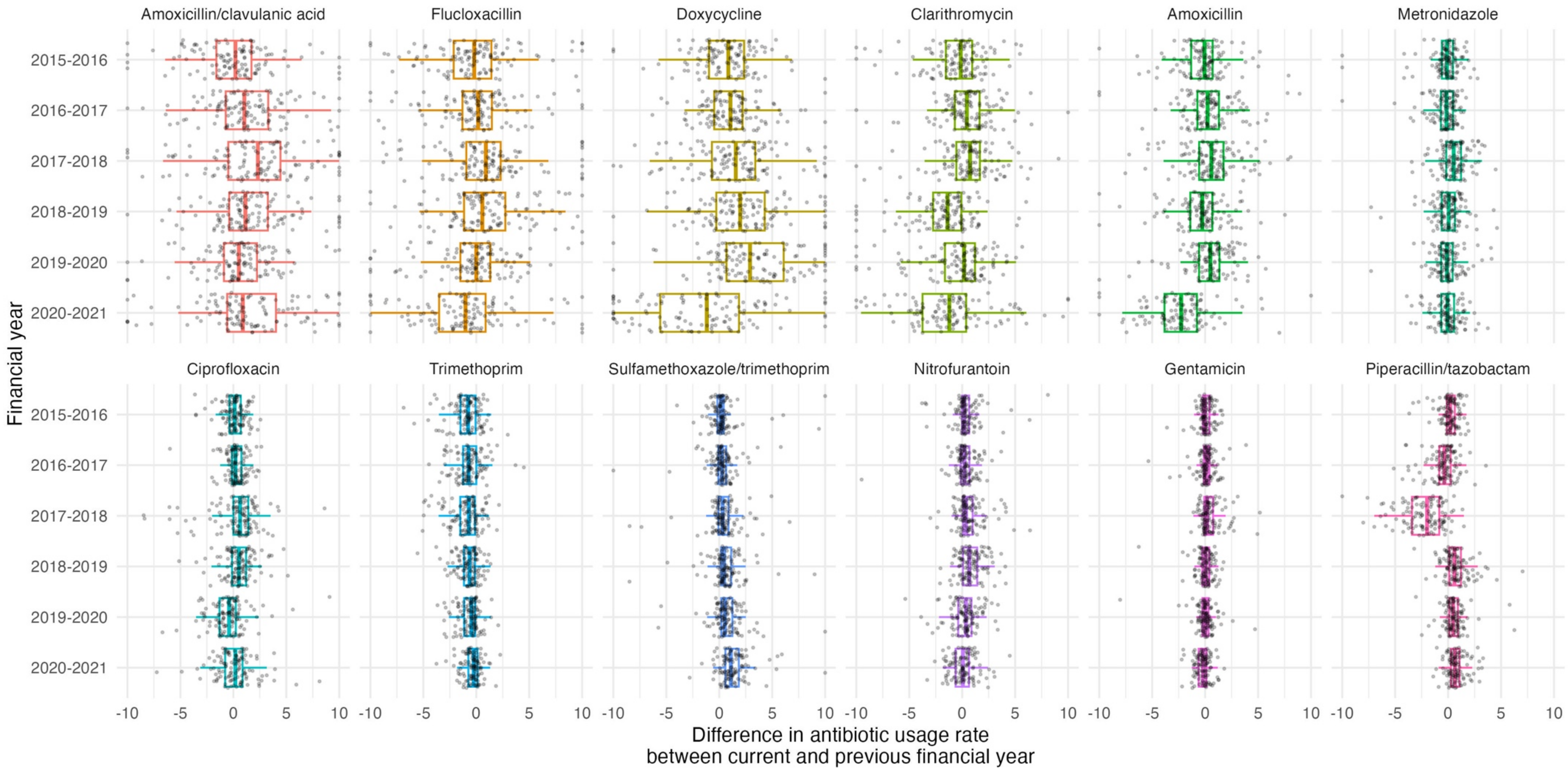

Note: one point per Trust-antibiotic. Note: Outliers outside of x-axis scale (absolute value >10) were truncated. Center line, median; box limits, upper and lower quartiles; whiskers, 1.5x interquartile range.

Supplementary Figure 9 Mean absolute error of predicting current resistance prevalence from previous value taken forwards by pathogen-antibiotic-FY over Trusts.

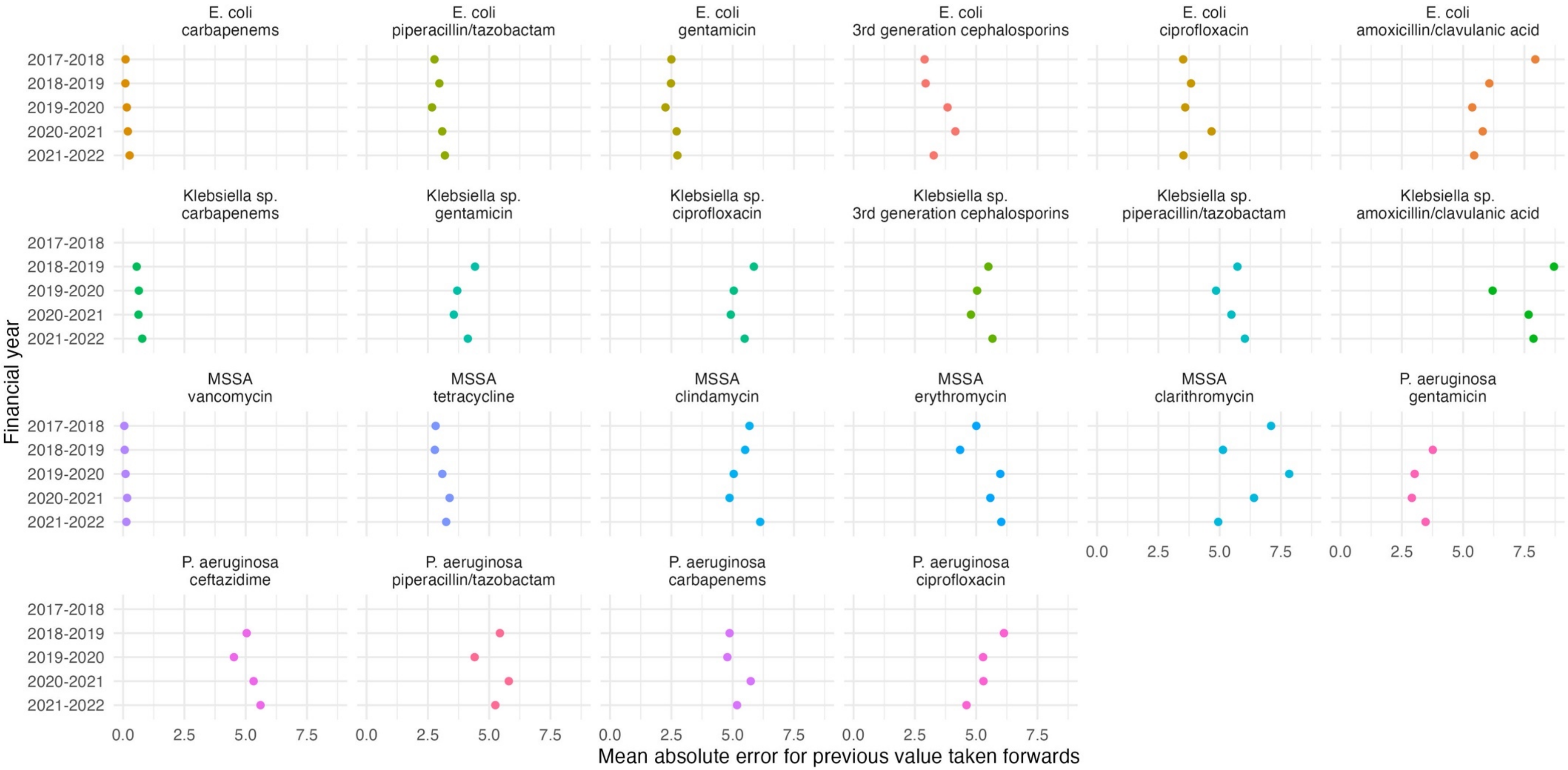

Supplementary Figure 10 Mean absolute error for prediction on test set (percentage resistance in FY2021-2022) for 6 different XGBoost prediction models: considering antibiotic usage in the previous year alone as input features (no information on previous resistance prevalence) and increasing the size of the training dataset by considering previous years as additional outcomes (XGBoost usage (1yr, "1yr double" and "1yr triple")), as well as XGBoost models with 3, 2 and 1 FY(s) historical data, for both usage and resistance (increasing the size of the training dataset by considering previous years as additional outcomes)

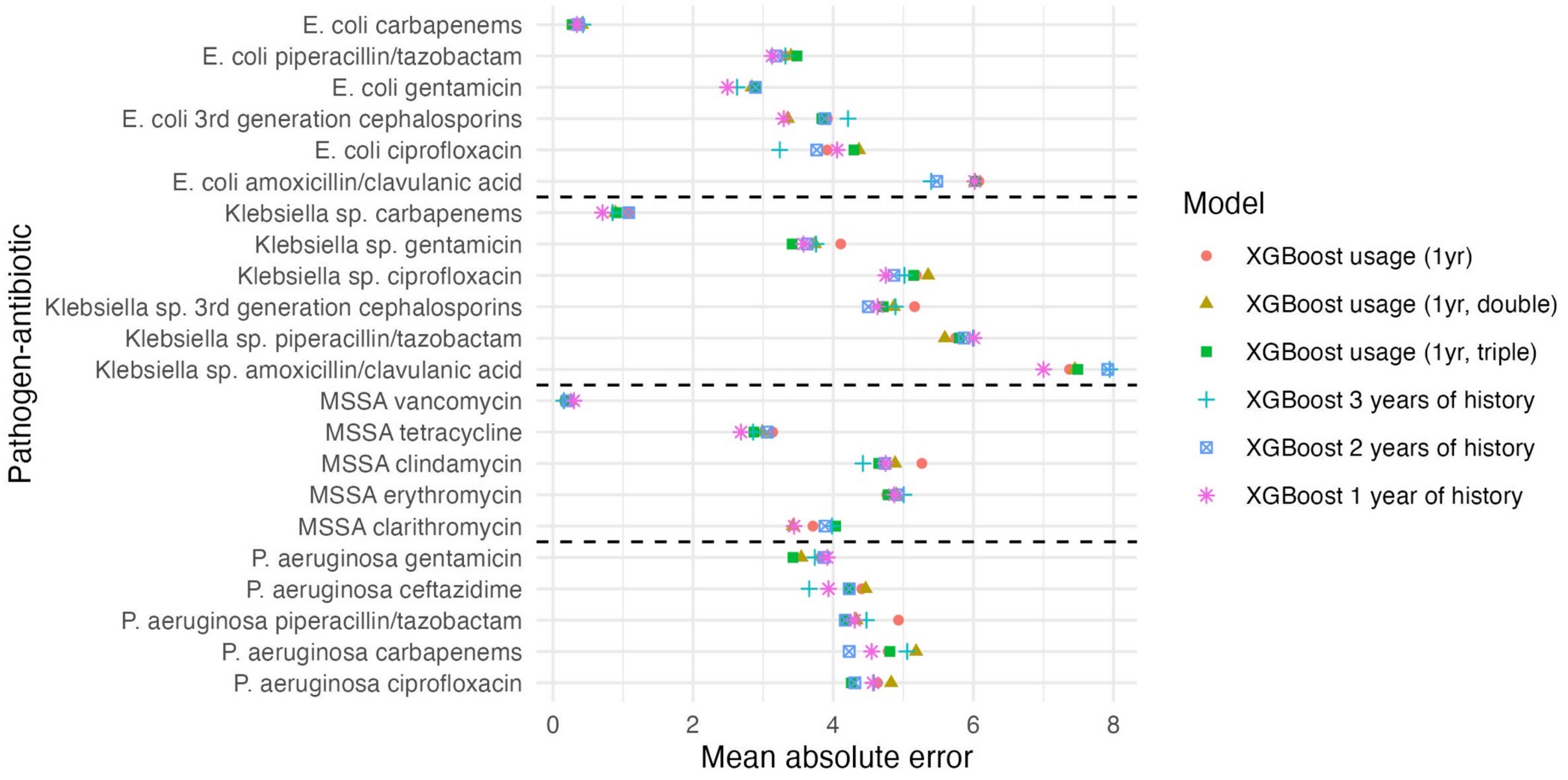

Supplementary Figure 11 Mean absolute error for prediction on test set (percentage resistance in FY2021-2022) for previous value taken forwards and 2 different XGBoost prediction models: XGBoost with all historical usage and resistance prevalence (XGBoost default), and XGBoost with selected features based on ranking according to mean absolute SHAP values being above that of an additional feature representing white noise (XGBoost default SHAP fs)

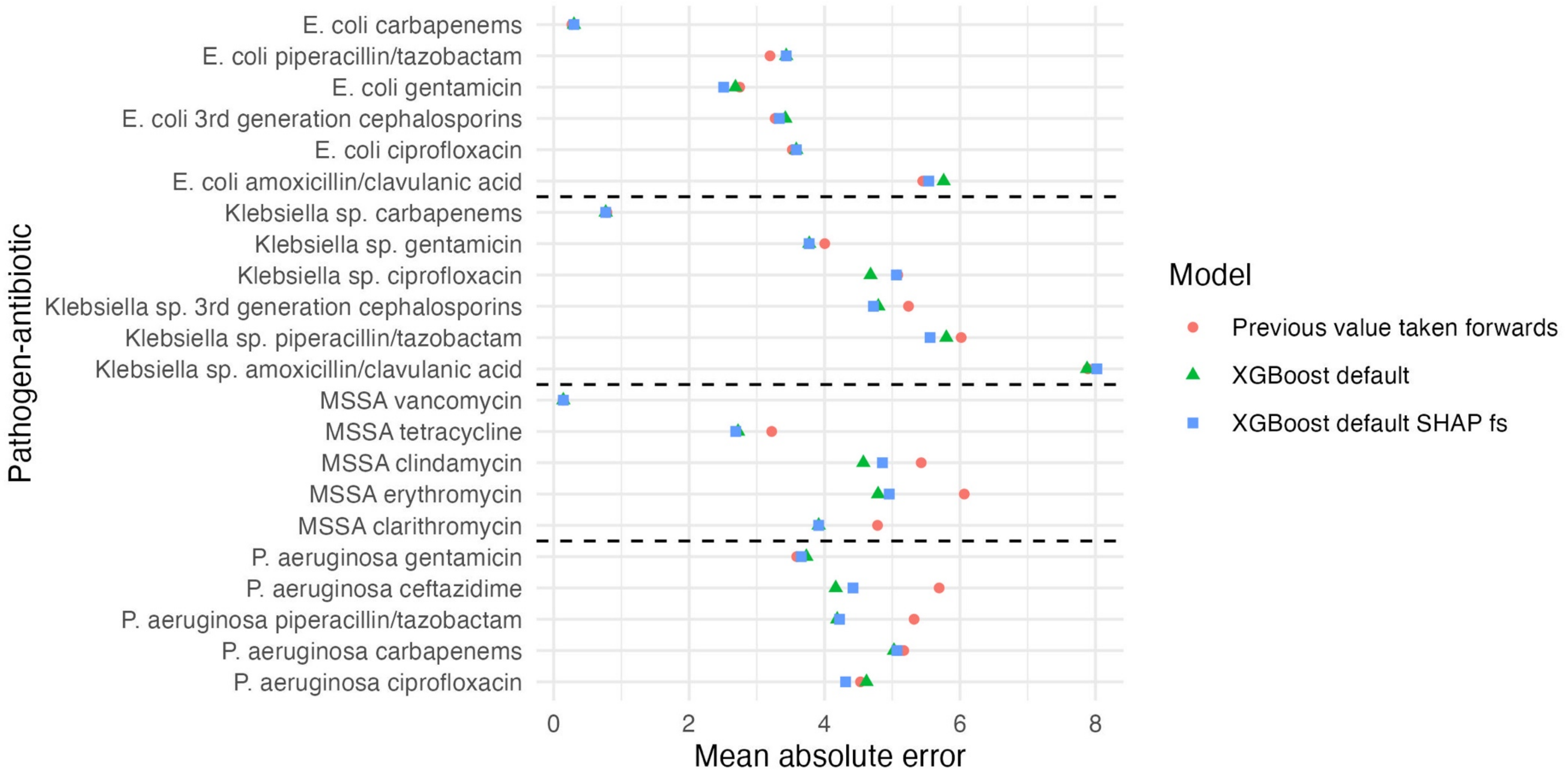

Supplementary Figure 12 Mean absolute error for prediction on test set (resistance prevalence in FY2021-2022) for 6 different prediction models split by absolute difference between FY2021-2022 and FY2020-2021 resistance prevalence >7.5% or ≤7.5% (A), >5% or ≤5% (B)

(A)

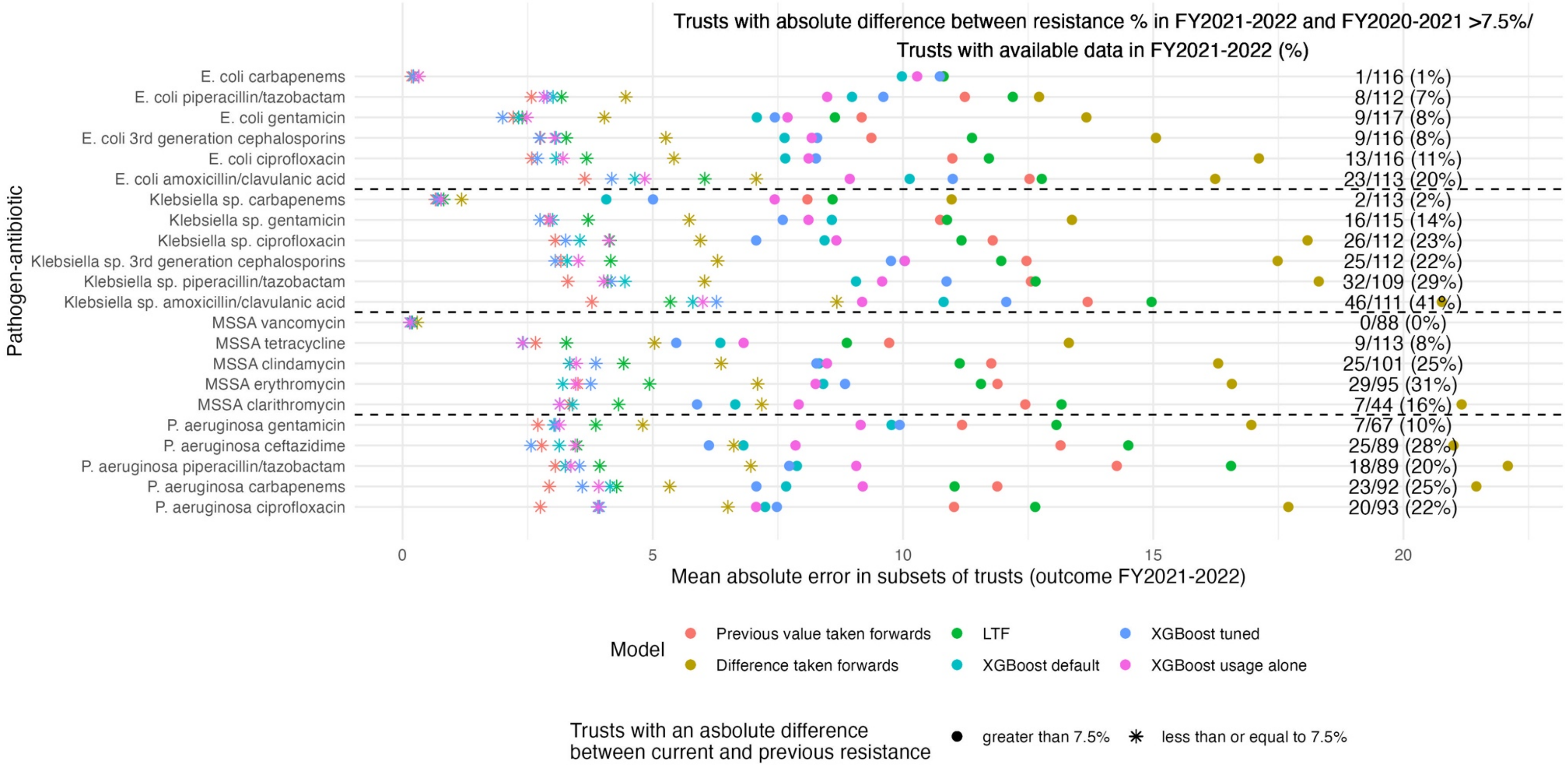

(B)

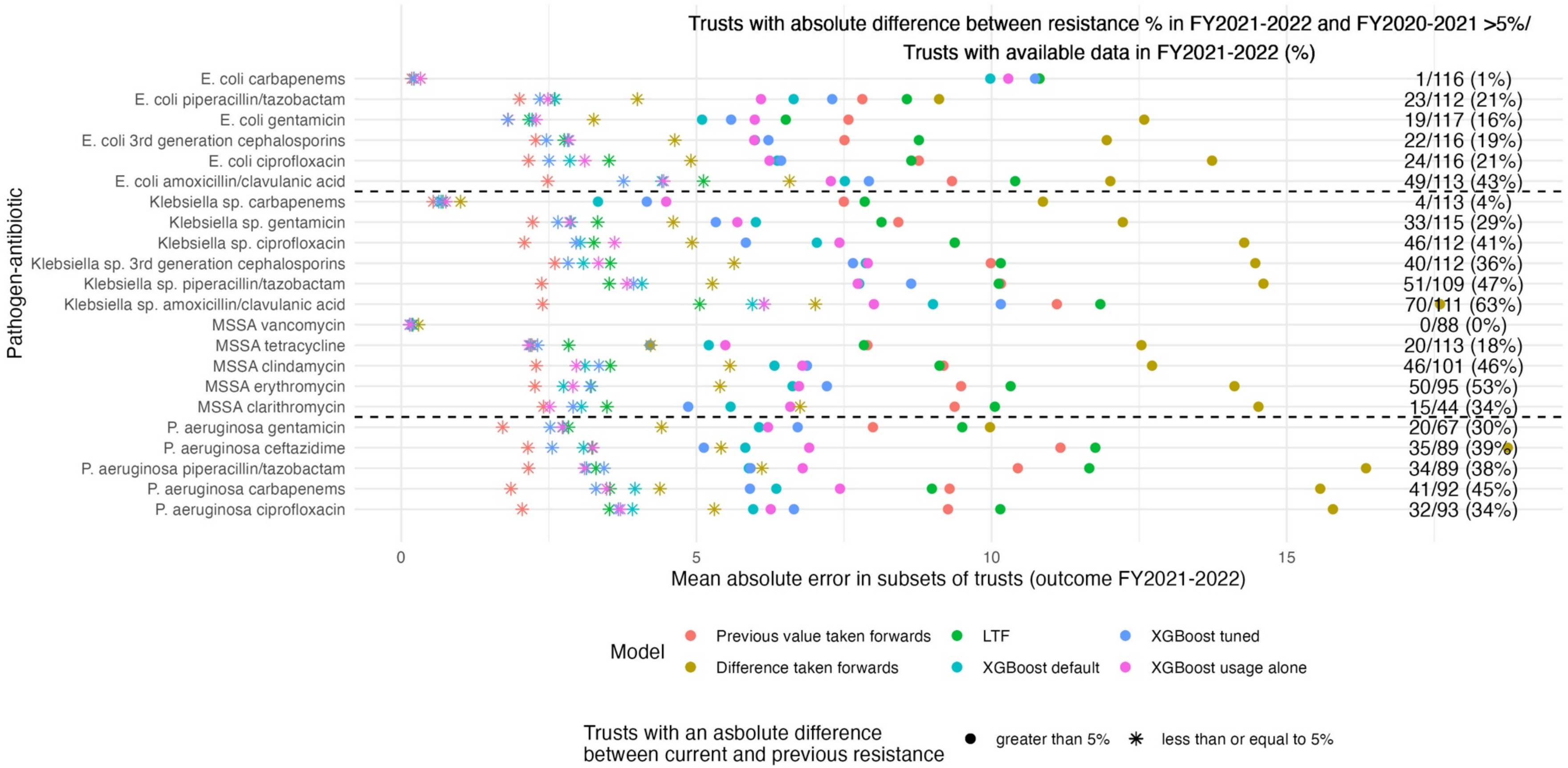

Note: 70 residuals that had either missing previous value or previous difference were excluded for comparability of performance measures between the models, although XGboost also made these predictions.

Supplementary Figure 13 Mean absolute error for prediction on test set (resistance prevalence in FY2021-2022) for 6 different prediction models in those Trusts with an absolute difference between FY2021-2022 and FY2020-2021 resistance prevalence >10%, split by whether the current resistance prevalence was greater or lower than the previous resistance prevalence

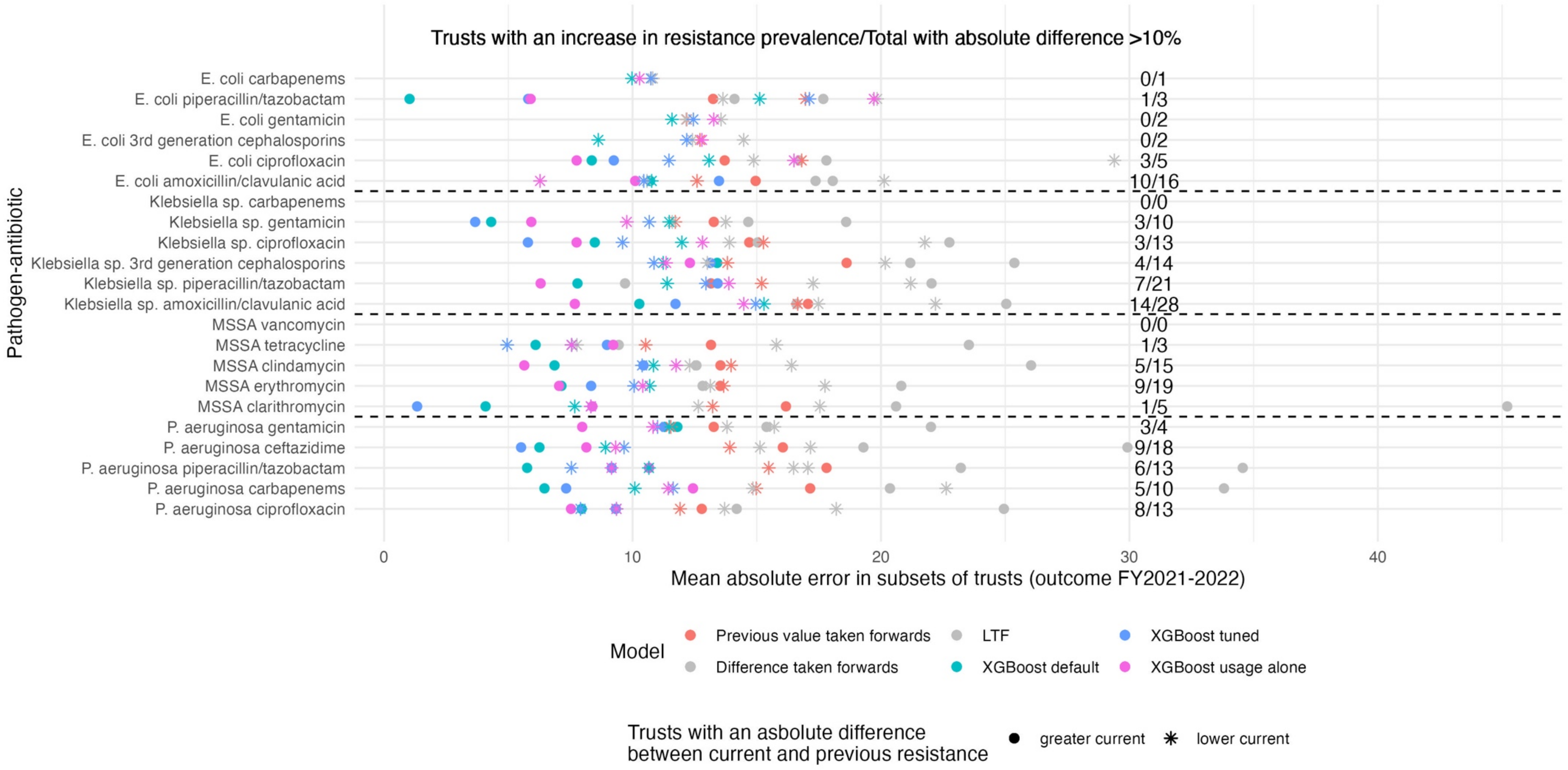

Supplement: Supplementary file 2 — Supplementary Information [file 43856_2024_606_MOESM2_ESM.pdf]
