# Supplementary material for: Predicting future hospital antimicrobial resistance prevalence using machine learning
Source: Commun Med (Lond). 2024 Oct 10;4:197. doi: 10.1038/s43856-024-00606-8 (PMC11467333; doi:10.1038/s43856-024-00606-8)
Supplement: Supplementary file 3 — Description of Additional Supplementary Files [file 43856_2024_606_MOESM3_ESM.pdf]

Description of Additional Supplementary Files

File name: Supplementary Data 1

File description: Number of Trusts contributing data (including zero usage rate) to each financial year per antibiotic together with mean and maximum usage rate across all Trust-FYs

File name: Supplementary Data 2 - 5

File description: corresponding to Figures 1-4
